# A Home-Based Mobile Health Intervention to Replace Sedentary Time With Light Physical Activity in Older Cancer Survivors: Randomized Controlled Pilot Trial

**DOI:** 10.2196/18819

**Published:** 2021-04-13

**Authors:** Cindy K Blair, Elizabeth Harding, Charles Wiggins, Huining Kang, Matthew Schwartz, Amy Tarnower, Ruofei Du, Anita Y Kinney

**Affiliations:** 1 Department of Internal Medicine University of New Mexico Albuquerque, NM United States; 2 University of New Mexico Comprehensive Cancer Center Albuquerque, NM United States; 3 School of Public Health Rutgers University Piscataway, NJ United States; 4 Rutgers Cancer Institute of New Jersey Rutgers University New Brunswick, NJ United States

**Keywords:** light-intensity physical activity, physical activity, sedentary behavior, mobile health, cancer survivors, consumer wearable, activity monitor, mobile phone

## Abstract

**Background:**

Older cancer survivors are at risk of the development or worsening of both age- and treatment-related morbidity. Sedentary behavior increases the risk of or exacerbates these chronic conditions. Light-intensity physical activity (LPA) is more common in older adults and is associated with better health and well-being. Thus, replacing sedentary time with LPA may provide a more successful strategy to reduce sedentary time and increase physical activity.

**Objective:**

This study primarily aims to evaluate the feasibility, acceptability, and preliminary efficacy of a home-based mobile health (mHealth) intervention to interrupt and replace sedentary time with LPA (standing and stepping). The secondary objective of this study is to examine changes in objective measures of physical activity, physical performance, and self-reported quality of life.

**Methods:**

Overall, 54 cancer survivors (aged 60-84 years) were randomized in a 1:1:1 allocation to the tech support intervention group, tech support plus health coaching intervention group, or waitlist control group. Intervention participants received a Jawbone UP2 activity monitor for use with their smartphone app for 13 weeks. Tech support and health coaching were provided via 5 telephone calls during the 13-week intervention. Sedentary behavior and physical activity were objectively measured using an activPAL monitor for 7 days before and after the intervention.

**Results:**

Participants included survivors of breast cancer (21/54, 39%), prostate cancer (16/54, 30%), and a variety of other cancer types; a mean of 4.4 years (SD 1.6) had passed since their cancer diagnosis. Participants, on average, were 70 years old (SD 4.8), 55% (30/54) female, 24% (13/54) Hispanic, and 81% (44/54) overweight or obese. Malfunction of the Jawbone trackers occurred in one-third of the intervention group, resulting in enrollment stopping at 54 rather than the initial goal of 60 participants. Despite these technical issues, the retention in the intervention was high (47/54, 87%). Adherence was high for wearing the tracker (29/29, 100%) and checking the app daily (28/29, 96%) but low for specific aspects related to the sedentary features of the tracker and app (21%-25%). The acceptability of the intervention was moderately high (81%). There were no significant between-group differences in total sedentary time, number of breaks, or number of prolonged sedentary bouts. There were no significant between-group differences in physical activity. The only significant within-group change occurred within the health coaching group, which increased by 1675 daily steps (95% CI 444-2906; *P*=.009). This increase was caused by moderate-intensity stepping rather than light-intensity stepping (+15.2 minutes per day; 95% CI 4.1-26.2; *P*=.008).

**Conclusions:**

A home-based mHealth program to disrupt and replace sedentary time with stepping was feasible among and acceptable to older cancer survivors. Future studies are needed to evaluate the optimal approach for replacing sedentary behavior with standing and/or physical activity in this population.

**Trial Registration:**

ClinicalTrials.gov NCT03632694; https://clinicaltrials.gov/ct2/show/NCT03632694

## Introduction

### Background

By 2030, there will be 22.1 million cancer survivors living in the United States, and two-thirds of them will be more than 65 years old [1]. Older cancer survivors are faced with both age- and treatment-related morbidity that increase their risk of physical function impairment and other comorbidities, including cardiovascular disease, diabetes mellitus, and osteoporosis [2-5]. These comorbidities further increase the risk of functional limitations. Compared with individuals without a history of cancer, cancer survivors have a 2- to 5-fold increased risk of having one or more functional limitations [5]. These chronic conditions are associated with diminished quality of life (QoL), premature death, and substantial financial costs [6-11]. Physical inactivity and sedentary behavior (too much sitting, which is distinct from too little exercise [12]) can increase the risk of or exacerbate these chronic conditions [13-19].

Recent research suggests that sedentary behavior has molecular and physiological effects distinct from a lack of exercise [20,21]. Sedentary behavior is defined as any waking behavior (ie, not sleep) characterized by minimal energy expenditure (≤1.5 metabolic equivalents [METs]) while in a sitting, lying, or reclining position [22]. Sedentary behavior is associated with an increased risk of cardiovascular disease [23,24], premature all-cause mortality [23,25-27], greater fatigue [28,29], and decreased physical function [11,29,30]. Furthermore, how sedentary time is accumulated throughout the day is important, as frequent short breaks in sedentary time can attenuate the negative physiological response associated with prolonged, uninterrupted periods of inactivity [31-34].

Among cancer survivors, less than 2% of waking hours are spent in moderate-to-vigorous physical activity (MVPA), up to 70% of waking hours are spent in sedentary activities, and the remaining time is spent in light-intensity physical activity (LPA) [35]. LPAs are associated with better physical health [36,37], including better physical function [37-40], reduced risk of incident disability [39,41], and better emotional well-being [36,40,42,43], independent of MVPA. The association between LPA and health outcomes is either only apparent or appears stronger in older adults and adults who are less physically active or have impaired lower extremity function [41,44-47]. Thus, disrupting and replacing sedentary time with LPA, rather than MVPA, are likely a more feasible approach to reducing sedentary behavior in older cancer survivors.

Behavior change interventions based on theory are generally more effective than atheoretical approaches [48-50]. Recent reviews suggest that goal setting, feedback, self-monitoring, problem solving, and social support are the most promising behavioral change techniques for interventions designed to reduce sedentary behavior [51-53]. Unlike simple pedometers, consumer wearable activity trackers include multiple behavior change techniques [54,55]. The ability to provide feedback in real time is particularly salient for sedentary behavior, as it is a largely subconscious behavior [51]. Furthermore, wearable activity trackers are readily available and low cost and, if effective, represent a scalable option for expanding the reach to a large number of cancer survivors, including in rural areas.

Given the deleterious effects of sedentary behavior on health, including cardiovascular disease and diabetes mellitus, conditions that are commonly observed in older cancer survivors, or for which they are at an elevated risk [56], the role of sedentary behavior in cancer survivorship has been identified as a research priority [35,57]. However, to date, few interventions have been designed to reduce sedentary time among cancer survivors [51]. Recently, several mobile health (mHealth) pilot or feasibility interventions have evaluated text messaging or wearable activity trackers as an intervention tool to decrease sedentary behavior in breast, prostate, and colorectal cancer survivors [58-60]. These interventions encouraged standing and stepping to replace sedentary behavior, with a primary focus on moderate-intensity activity. Preliminary results suggest that mHealth interventions are feasible and acceptable in this population and have the potential to replace sedentary behavior with physical activity, at least in the short term. However, additional research is needed to further evaluate effective strategies to reduce sedentary time by either replacing it with standing, stepping, or both.

### Objectives

The purpose of this study is to examine the feasibility, acceptability, and preliminary efficacy of an mHealth intervention for disrupting (frequent breaks) and replacing sedentary time with intermittent bouts of LPA (standing and stepping). The 13-week intervention used the Jawbone UP2 activity monitor and associated smartphone app to promote awareness and enable self-monitoring of both physical activity and inactivity. We evaluated 2 versions of the mHealth intervention: a low-touch approach providing only tech support and a higher resource approach that included health coaching in addition to the tech support. This would allow us to determine whether a low-cost, consumer-based technology (wearable activity tracker plus smartphone app) is effective in meeting the goals or whether health coaching is needed to cover additional behavior change techniques not provided in the wearable activity tracker. Our primary objective is to determine the feasibility and acceptability of the 2 versions of the mHealth intervention by assessing recruitment, retention, and adherence rates; monitoring adverse events; and evaluating satisfaction with the program. In addition, we examined the preliminary efficacy of the intervention on changes in objective measures of daily total sedentary time and the number of breaks in sedentary time. Our secondary objective is to explore changes in objective measures of physical activity, physical performance, and self-reported QoL.

## Methods

### Study Design

This study was a 3-arm pilot randomized controlled trial (RCT). Older cancer survivors were randomized in a 1:1:1 allocation to the tech support intervention group, the tech support plus health coaching intervention group, or a modified waitlist control group. The intervention used a consumer wearable activity tracker (Jawbone UP2 wristband) that was paired with a smartphone app to promote awareness and enable self-monitoring of both inactivity (band gently vibrates after a specified time of inactivity) and physical activity (eg, steps per day). We evaluated 2 versions of the intervention: a low-touch approach providing only tech support and a higher resource approach that included health coaching in addition to the tech support. Each intervention group was compared with the waitlist control group. Recruitment for the trial began in June 2016, and data collection was completed in July 2017.

### Eligibility

Eligibility criteria for the feasibility study included (1) men and women aged 60 years and older (reduced from 65 years to increase the number of participants who own a smartphone); (2) those who were diagnosed as having an invasive, local or regionally staged cancer within the past 7 years (time frame increased the likelihood that address and phone number in cancer registry were still current) and completed primary treatment (surgery, radiation, and chemotherapy); (3) those who owned a smartphone capable of running the Jawbone UP2 smartphone app; (4) those who were willing to be randomized to any of the 3 study arms, attend 2 clinic visits, and wear activity monitors; (5) those who were able to read, speak, and understand English; (6) those who were living independently and were capable of walking 3 blocks (approximately 1/4 mile or 1300 steps) without an assistive device (eg, cane and walker); (7) self-reported sedentary time (during waking hours) of ≥6 hours/day (Longitudinal Aging Study Amsterdam Sedentary Behavior Questionnaire: hours and minutes in a day spent in 10 activities, on average, during a weekday [61]); (8) those who were not currently participating in a program to decrease sedentary time or increase physical activity and not currently using a fitness tracker; (9) those who had no paid employment or volunteer position for more than 20 hours per week (to avoid potential confounding by occupational activity/inactivity); (10) those who had no severe impairments (in seeing or hearing) or preexisting medical limitations for engaging in daily LPA (eg, severe orthopedic conditions, pending hip/knee replacement, dementia, and oxygen dependent); (11) those who had residence within 60 miles of the research clinic (to reduce travel burden and improve retention and compliance); and (12) those who had a wrist size of 14 cm to 20 cm to wear the Jawbone UP2 activity wristband during the intervention. Individuals who met the physical activity guidelines (150 minutes per week of MVPA) [17,62] were eligible because sedentary behavior is a risk factor for morbidity and mortality independent of MVPA.

### Recruitment

The population-based New Mexico Tumor Registry, a founding member of the Surveillance, Epidemiology, and End Results Program [63], was used as the primary source for identifying potential study participants. Additional sources included posting flyers at selected locations, including senior centers and libraries. After identifying potentially eligible study participants, the New Mexico Tumor Registry mailed a letter that introduced the study and gave potentially eligible participants the opportunity to decline further contact. Contact information for individuals not refusing further contact was provided to the study team after a 3-week waiting period. Potential participants were then mailed a letter explaining the study and a consent form. One week later, the staff telephoned to discuss the study, answer questions, begin the consent process, and verify eligibility. Up to 3 attempts (later expanded to 4) were made to reach individuals who had a valid telephone number. A written informed consent for the interested and eligible participants was obtained during the baseline clinic visit.

### Randomization

After a 1-week run-in period, a member of the research team opened the next sequentially numbered sealed envelope (created by a biostatistician) to reveal the randomization status. Participants were block randomized with equal allocation to 3 arms (tech support, tech support plus health coaching, or modified waitlist control) according to obesity status (BMI <30 vs ≥30 kg/m^2^).

### mHealth Intervention

#### Theoretical Framework

The theoretical framework used to guide this intervention was the social cognitive theory [64,65]. The intervention primarily targeted the theoretical constructs of knowledge, behavioral skills, behavioral capability, and self-efficacy. Wearable activity trackers, such as Jawbone, include a number of behavioral change techniques associated with decreasing sedentary behavior and increasing physical activity (eg, goal setting, graded tasks, and self-monitoring) [54,55]. However, some of the key techniques are missing and were supplemented with educational materials and technology support. Additional behavior change techniques were provided by the health coaches for the health coaching intervention, such as the identification of barriers and problem solving. Health coaches also provided encouragement and support and encouraged positive support from family and friends. A list of the behavior change techniques, theoretical constructs, and examples of strategies to promote behavior change in this mHealth intervention is presented in Table 1.

**Table 1:** Behavior change techniques and strategies to promote behavior change via educational materials, the Jawbone tracker and app, or tech support coaching or health coaching.

Behavior change technique	Theoretical construct	Examples of strategies	TS^a^ group				HC^b^ group		
			EM^c^	JB^d^	TS	EM		JB	HC
Information on consequences of behavior	Knowledge	Educational materials on harms of physical inactivity and sedentary behavior; also discussed with health coach	✓^e^			✓			✓
Goal setting (behavior)	Behavioral skills; self-efficacy	Set weekly short-term and long-term step goals; tech support for changing goal settings on app; idle alert goal (every 30 min) and step goal (graded increase in steps)		✓	✓			✓	✓
Barrier identification and problem solving	Barrier self-regulatory efficacy	Work with health coach to assess barriers and identify solutions to breaking up sedentary time and getting more steps throughout the day							✓
Set graded tasks	Self-efficacy	Encourage incremental and achievable sedentary (breaks) and step goals		✓				✓	
Review of behavioral goals	Behavioral skills	Using Jawbone app to review daily progress and weekly patterns for longest idle time and steps		✓				✓	
Generalization of a target behavior	Behavioral capability	Educational materials with suggestions for breaking up sedentary time in different ways and locations; additional support from health coach	✓			✓			✓
Self-monitoring of behavior	Behavioral skills	Using Jawbone app to review daily progress and weekly patterns and provide immediate feedback (idle alert and longest idle time)		✓				✓	
Feedback on behavior	Behavioral skills	Jawbone tracker and app provide immediate feedback; health coach to discuss whether goals were met		✓				✓	✓
Information on where and when to perform behavior	Behavioral capability	Education materials to suggest tips for disrupting SB^f^; Jawbone idle alert to prompt when to stand up and move	✓	✓		✓		✓	
Instructions on how to perform the behavior	Self-efficacy; behavioral skills	Print materials and coaching provide instructions on setting up and using the Jawbone tracker and app	✓		✓	✓			✓
Social support	Social support	Health coach provides support and encouragement; provide information and suggestions when asked; encourage enlisting positive support from family members and friends to take more steps throughout the day							✓
Use prompts/cues; prompt practice	Cues to action	Jawbone idle alert will prompt user to disrupt sitting with standing or stepping; Jawbone alerts will prompt more steps to reach daily goal		✓				✓	

^a^TS: tech support.

^b^HC: health coaching.

^c^EM: educational material.

^d^JB: Jawbone tracker and app.

^e^Primary source for the behavior change technique.

^f^SB: sedentary behavior.

#### Components of the Intervention

The mHealth intervention consisted of educational materials; a Jawbone (in)activity tracker; a free, commercially available smartphone app; and support via 5 telephone calls. The only difference between the 2 intervention groups was the level of telephone support. One group received only support related to the use of technology (tracker and app, tech support group), whereas the other group received additional health coaching to meet the study goals (tech support plus health coaching group).

#### Educational Materials

Upon randomization, both intervention groups received brief educational materials by mail. These materials explained the negative consequences of sedentary behavior, especially prolonged periods of sitting, and included suggestions for how to disrupt and replace sedentary time with LPA. Examples of suggestions provided included walking around the house during television commercial breaks, standing while talking on the telephone, and parking the car further away from the entrance [66]. The summary graph representing the most active and least active days from the week-long collection of objectively measured sedentary time, standing, and stepping (output from the activPAL3 monitor) was mailed to study participants (for later discussion with their coach; Multimedia Appendix 1). The waitlist control group received educational materials at the postintervention follow-up when they received their activity tracker and smartphone app.

#### Jawbone UP2 Activity Tracker

Upon randomization to either of the 2 intervention groups, participants were mailed the Jawbone UP2 activity wristband and provided detailed instructions for installing the free, commercially available app on their smartphone and for using the wristband with the app. At the time the study was designed (2015), this was one of the few consumer wearable activity trackers that had the ability to alert the wearer after a specified time of inactivity. For the Jawbone monitor, this feature was known as an *idle alert*, which notified the user of inactivity via a gentle vibration of the wristband (eg, users select time in increments of 15 minutes). The assigned coach telephoned participants to assist with the installation and setup of the activity tracker and smartphone app.

**Figure 1:**
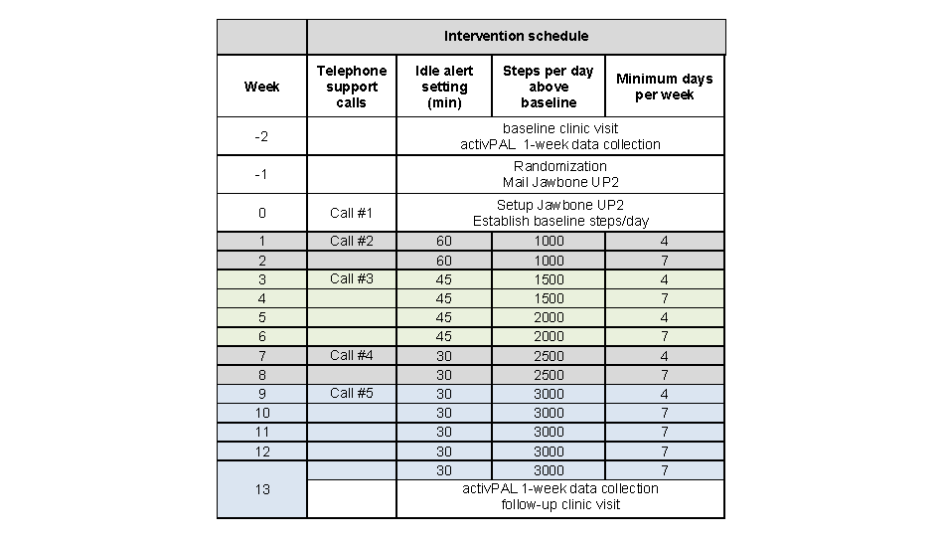
Weekly schedule for the tech support and health coaching intervention groups.

The goal was to decrease daily total sedentary time and increase the number of breaks in sedentary time by replacing/disrupting sedentary time with intermittent bouts of LPA (standing and stepping). The key message for the activity prescription was to “sit less, stand more, and move more, throughout the day, every day.” This message was included in the educational materials and was repeated during each of the 5 support telephone calls. Participants were encouraged to stand up and move at least once every 30 minutes. To encourage more movement than standing, participants were provided with a graduated steps per day goal of adding 3000 steps per day above their baseline level by week 9 (schedule in Figure 1). This target represents approximately 40 extra minutes of leisurely paced walking [67] and is associated with health benefits [36,68]. When combined with 20 minutes of standing, this would result in replacing 1 hour of sedentary time with 1 hour of LPA per day. A minimum intensity and a minimum bout duration for stepping were not provided, thus allowing the participant to self-select how to accumulate their extra daily steps.

The participants were instructed to wear the Jawbone during waking hours and were encouraged to track their activity at least once a day by viewing their results on the app. A commercially available app was used without any modifications by the research team. The app included a daily summary of total steps, total and longest active time, and longest idle time (longest time spent sedentary). To promote gradual and sustained change in LPA, participants were asked to increase the number of steps per day (above their individual baseline level), during weeks 1 to 9, and then work to maintain their goal during weeks 10 to 13 (Figure 1). Similarly, the *idle alert* setting began at 1 hour, decreased to 45 minutes, and then every 30 minutes. Participants in both intervention groups received guidance from their coaches on how to change the settings in their app.

#### Tech Support and Health Coaching Calls

The coaches were graduate students who received study-specific training, including 4 practice calls with staff members before calls to study participants. One coach was assigned to each intervention group participant based on their type of phone, for example, iPhone vs android or other mobile operating system. Phone scripts were used to guide the coaches to deliver only tech support versus tech support plus health coaching. During the first telephone call (week 0; Figure 1), coaches helped the participants to set up their Jawbone monitor. During the second telephone call (week 1), each coach reviewed the activPAL3 baseline summary data (total and percentage of time spent sedentary, standing, and stepping for best and worst days) with the participant and discussed the importance of reducing sedentary time, especially prolonged periods of inactivity. Additional telephone calls (15-20 minutes) were made during weeks 3, 7, and 9 to verify completion or to assist participants with changing the steps per day goal and *idle alert* setting on their app (if needed). Tech support coaches provided support related only to the technology (Jawbone UP2 activity tracker and/or smartphone app), including troubleshooting technical issues. In contrast, health coaches provided additional support to help their participants identify a list of LPAs to replace/disrupt sedentary time and to achieve the ≥3000 steps per day goal, review the importance of goal setting and self-monitoring, and help troubleshoot problems and find solutions to meet their goals.

#### Problems With Jawbone UP2 Monitors

During the intervention, the Jawbone UP2 wristbands started to fail (ie, losing settings, losing connection with app, and not syncing data), affecting 13 of 36 intervention group participants. New Jawbone UP2 wristbands were purchased by the study team through other sources (Amazon website), but many of these wristbands also failed. We were able to buy and test UP2 wristbands to replace the failed units for the intervention group participants. Given these major issues and lack of support from Jawbone, waitlist control participants enrolled later in the study were provided with a Fitbit Alta (Fitbit Inc) at the end of the 13-week study. This product was similar to the Jawbone UP2 in that it provided an inactivity alert (reminder to move every hour) and allowed the user to set a step goal and track their steps.

#### Waitlist Control Group

Upon completion of the study, the control group received a shortened version of the intervention, that is, education materials, tracker, and smartphone app, and instructions for use to track their activity/inactivity. During the postintervention clinic visit, a study team member helped the participant to install the app on their smartphone; pair the tracker to their phone; and select settings for the idle alert and step goal. Each participant in this group was also offered up to 2 telephone calls with one of the coaches to receive tech support or other support to meet their personal goals for reducing sedentary behavior and increasing their activity via steps.

### Procedures

#### Baseline Assessment

Pre- and postintervention clinic visits were conducted at the University of New Mexico Clinical and Translational Science Center. Assessments were conducted primarily by study team members not involved in intervention delivery; however, occasionally, there was overlap owing to limited resources. The baseline assessment included obtaining written informed consent, simple anthropometric measurements (height and weight), and objective physical function measures (physical tests of lower extremity function and mobility). At the end of the visit, study participants were instructed on how to attach the activPAL3 research-grade activity monitor and then observed to verify correct placement. Participants were instructed to wear the activPAL3 monitor for 24 hours/day for 1 week and on how to remove and return (via self-addressed stamped mailer) the monitor to study staff at the end of that week.

#### Follow-Up Assessment

At the end of the intervention, the activPAL3 research-grade monitor, attachment supplies, and instructions were mailed to all participants to collect 1 week of sedentary behavior and physical activity data. The project manager called to review the instructions for use and answer any questions. Additional postintervention outcome measures were collected at the clinic visit at the end of week 13. Participants received US $50 gift cards to complete the baseline and follow-up assessments and to help cover the costs of accessing the app on their smartphone. In addition, participants were allowed to keep the Jawbone UP2 activity tracker at the end of the study.

#### Device-Based Measures

Sedentary behavior and physical activity were measured using an activPAL3 research-grade monitor (PAL Technologies Ltd). activPAL3 is a lightweight device worn on the thigh and includes both an inclinometer (to detect changes in position) and a triaxial accelerometer. activPAL is the gold standard in sedentary behavior research and provides accurate measures of sitting (or lying), standing, and stepping [69-72]. Participants wore the device for 24 hours per day for 7 days, before and after the intervention. The device was only removed for bathing or swimming or if an adverse reaction occurred to the Tegaderm dressing used to attach the device. Participants recorded in their diary, the day/time when the device was attached, each time it was removed and reattached, and the time they went to bed at night and woke up in the morning.

### Outcomes and Measurements

#### Feasibility and Acceptability Outcomes

The feasibility and acceptability of the mHealth intervention were determined by achieving the following goals: (1) to recruit 60 older cancer survivors; (2) to retain 80% of the sample; (3) to achieve 80% adherence to the intervention; (4) to have no serious adverse events attributable or possibly attributable to the intervention, defined as any condition that is life threatening and results in overnight hospitalization or a physical or cardiac event serious enough to require medical attention; and (5) to achieve high satisfaction (acceptability) rates with the intervention; to have 75% or more of participants report agree or strongly agree on a 5-point Likert scale.

Retention was calculated as the percentage of participants who completed the follow-up clinic visits and accelerometer assessment. Adherence to wearing the Jawbone UP2 tracker, checking the app daily, and acting on the *idle alert* was assessed with 4 questions. Response items included never, rarely, sometimes, often, or very often. For adherence to the intervention, we calculated the percentage of intervention group participants who responded *often* or *very often* to the 4 questions regarding their use of the Jawbone tracker and app. In addition, the completion of telephone support calls was tracked. Acceptability and evaluation of the Jawbone UP2 technology (UP2 tracker and app) were assessed using 7 questions. Response items included strongly disagree, disagree, neutral, agree, or strongly agree. For acceptability, we calculated the percentage of respondents who responded *agree* or *strongly agree* to the 7 questions regarding ease of use, motivation, intention for continued use, and recommendations of this technology. Adherence and acceptability were stratified based on whether participants received a replacement Jawbone tracker owing to severe malfunctioning.

#### Primary Preliminary Efficacy Outcomes

The primary behavioral outcomes of interest were changes in total sedentary time (average minutes per day) and number of breaks from sitting (average breaks per day). As the opportunity to interrupt sitting while standing or stepping is dependent on the amount of sedentary time, the break ratio was also calculated. The break ratio was defined as the number of absolute breaks divided by total sedentary time.

### Secondary Preliminary Efficacy Outcomes

#### Device-Based Measures of Sedentary Behavior and Physical Activity

activPAL was also used to assess changes in total minutes spent in prolonged sedentary bouts, minutes per day spent standing, number of steps per day, and minutes of light- and moderate-intensity physical activity (reported separately). A prolonged sedentary bout was defined as 30 or more continuous minutes in a seated or lying position [73]. LPA was defined as stepping at a cadence equivalent to 1.5 to 3.0 METs [73]. A MET is a multiple of resting energy expenditures. With resting (sitting quietly) energy expenditure defined as 1 MET, a 3-MET activity expends the energy of rest by 3 times, whereas a 5-MET activity expends the energy of rest by 5 times. Standing is also considered an LPA and has been reported separately from light stepping. Moderate-intensity physical activity was similarly defined, but with MET values from 3.0 to 5.9. Vigorous-intensity physical activity was defined as MET values of ≥6.0 or higher. As the guidelines at the time this intervention were designed specified that MVPA be accumulated in minimum bouts of 10 minutes, we also evaluated guideline bouts of MVPA [17,62]. The activPAL monitor provides accurate and precise categorization of sedentary time, LPA, and MVPA in a free-living setting (96.2% accuracy compared with direct observation) [73].

#### Objectively Measured Physical Performance

The emphasis on frequent interruptions of sedentary behavior with standing and stepping has the potential to improve lower extremity physical function. This was measured using the Short Physical Performance Battery (SPPB). The SPPB includes tests of standing balance, walking speed (timed 8-ft walk at usual speed), and lower body strength (time to rise from a chair 5 times) [6,74]. Scores range from 0 (not attempted) to 4 (highest score) for each test, with a total score ranging from 0 to 12. This battery has strong predictive validity and is responsive to changes [6,74].

#### Subjective Measures

Given the inverse association reported between sedentary behavior and QoL [29,75,76], we evaluated changes in QoL as a secondary outcome. The Medical Health Outcomes Study Short Form 36-item survey (SF-36, version 2) was used to assess health-related QoL. The SF-36 includes 8 individual scale scores and 2 component summary scores for physical and mental health and well-being. This instrument is valid and reliable for use in healthy and chronically ill adults [77,78]. Surveys were scored using QualityMetric [79]. Raw scores range from 0 to 100, with higher scores indicating better functioning and well-being. T-scores represent a linear transformation, normed to the US population, with a mean of 50 (SD 10). Pain and fatigue were assessed using the patient-reported outcomes measurement information system (PROMIS) Pain Interference Short Form 8A and the functional assessment of chronic illness therapy (FACIT)-Fatigue scale (version 4) [80,81]. The pain interference survey included 8 questions on whether and the degree to which pain interfered with various activities during the past 7 days. The fatigue scale included 13 questions on whether fatigue affected a person’s life during the past 7 days and the degree to which fatigue affected a person’s life during the past 7 days.

#### Other Measures

In addition, sociodemographics, cancer-related data, comorbidities, and simple anthropometrics were ascertained via paper surveys to characterize the study population. Sociodemographic data were assessed via questionnaires at baseline, including age, sex, race/ethnicity, education, income range, and marital status. Smoking status (current, former, or never smoker) was also assessed at baseline. Cancer data were obtained from the New Mexico Tumor Registry (cancer type, stage, and date of diagnosis) and from self-reported surveys (treatment [yes/no]: surgery, chemotherapy, radiation, hormone therapy, and date primary therapy completed). The Self-Administered Comorbidity Questionnaire [82] was used to assess the number of conditions and their impact on usual activities. The number of comorbidities and whether they limited activities were summed and categorized as 0 or 1 comorbidity (activities not limited), 1 comorbidity (activities limited), and 2 or more comorbidities (activities limited). Height (nearest 0.5 cm) was measured at the baseline clinic visit. Weight (nearest 0.1 kg) was measured at both the baseline and follow-up clinic visits. BMI (kg/m^2^) was calculated and categorized as normal weight (18.5 kg/m^2^-24.9 kg/m^2^), overweight (25.0 kg/m^2^-29.9 kg/m^2^), and obese (≥30 kg/m^2^).

### Data Processing and Statistical Analysis

#### Processing of activPAL Data

activPAL3 data were downloaded using activPAL software (version 7; PAL Technologies Limited). The event files (start/stop time for sitting/lying, standing, and stepping) were processed using the activPALProcessing R package (version 1.0.2) [73,83]. After converting the event file into a second-by-second data file (second-by-second R function), other R functions were used to calculate the sedentary behavior and physical activity metrics. Only days with 10 or more hours of wear per awake time were included, and only the first 7 valid days were included (extra days were excluded). To be included in the analyses, a participant needed at least one valid day of activPAL3 data from baseline, which is consistent with the intention-to-treat principle and similar to other recent trials [58,84]. Owing to the large variability in the within- and between-person average number of awake per wear hours, all activPAL metrics were standardized to a 15-hour awake per wear day (average in this study sample). Additional details of the activPAL data collection and processing are included in Multimedia Appendix 2 [69-73,83,85], similar to other studies [59,85].

#### Efficacy Outcomes

Baseline descriptive characteristics (mean, SD or frequency, %) were used to characterize the study population. Intent-to-treat analyses were conducted to evaluate changes in sedentary behavior metrics and secondary outcomes. Linear mixed methods were used to estimate the within- and between-group differences for each outcome. Each model included a fixed effect for group (tech support, health coaching, and waitlist control), time (before and after the intervention), and group by time interaction. A subject-level random effect was included to account for the correlation between repeated measurements of the same individuals over time. Statistical analyses were performed using SAS (version 9.4) and R (v.3.4.3).

Complete case analyses were conducted that only included individuals with complete data (12 tech support, 17 health coaching, and 18 controls). A sensitivity analysis was conducted that excluded individuals with fewer than 4 valid days of activPAL data (3 participants from the tech support only group). In addition, a sensitivity analysis was conducted by excluding the 12 intervention participants who experienced major problems with their Jawbone tracker (ie, required 1 or more tracker replacements, excluding 6 participants in each intervention group). For this sensitivity analysis, the control group was restricted to control participants who completed their baseline visit during the same period as the intervention participants, to account for potential seasonality effects (ie, before mid-February 2017, excluding 6 controls).

The proposed pilot intervention was a feasibility and acceptability intervention and thus was not powered to detect small effect sizes for change in any outcome. However, for sedentary time, with 20 people per group, assuming a 2-sided alpha level of 0.05 and an SD of 1.4 hours, there was 80% power to detect a difference of 1.3 hours in sedentary time between 2 groups [86,87].

## Results

### Feasibility

**Figure 2:**
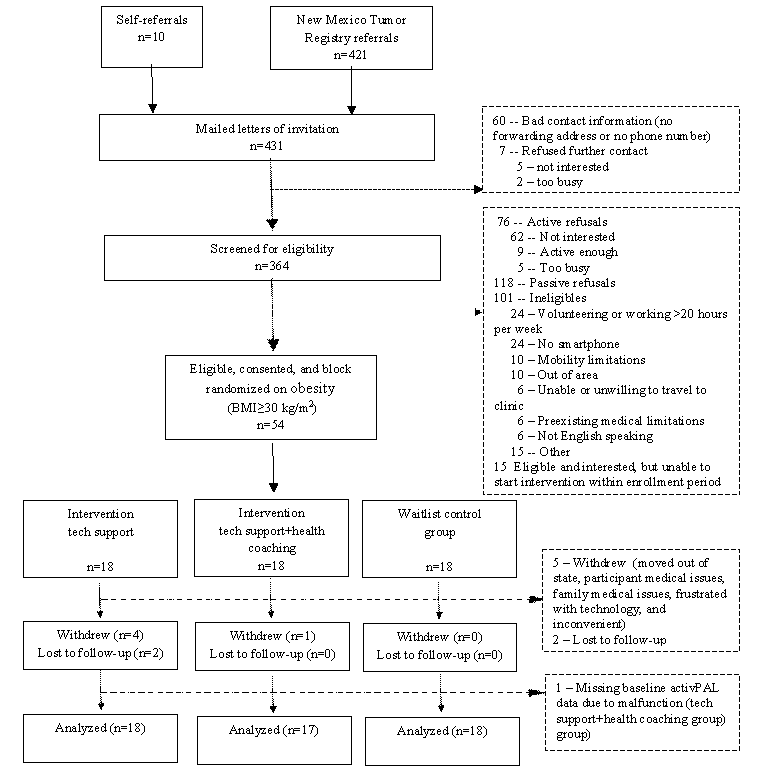
CONSORT (Consolidated Standards of Reporting Trials) diagram.

The New Mexico Tumor Registry identified 421 potentially eligible participants and, after accounting for a 3-week opt-out period, forwarded contact information on 354 individuals to study staff. Of the 364 individuals (including 10 self-referrals) we attempted to contact by telephone, 76 refused to participate, 101 were ineligible, and 118 were considered passive refusals after 3 to 4 attempts to contact via telephone (Figure 2; see Multimedia Appendix 3 for CONSORT [Consolidated Standards of Reporting Trials] checklist). The overall response rate was 20.5%. The top 3 reasons for ineligibility included not owning a smartphone, volunteering or working for more than 20 hours per week, and mobility limitations. The top 2 reasons for refusal included a lack of interest and feeling that they were already active enough. An additional 15 individuals were eligible and interested but were unable to begin the intervention before the end of the enrollment period. Owing to the major malfunctions with the Jawbone UP2 monitors during the second half of the study, enrollment was stopped early with a final enrollment of 54 participants.

Retention in this 13-week intervention for older cancer survivors was moderately high (47/54, 87%). All of the dropouts occurred in the intervention groups, with the majority in the tech support group (6 of 7). The reasons included personal or severe family illness (n=2), move out of state (n=1), inconvenience (n=1), frustration with technology (n=1), and loss to follow-up (n=2). Notably, 3 of the 7 dropouts occurred among individuals who experienced malfunctioning with their Jawbone monitor (tech support group). Individuals who dropped out or were lost to follow-up were more likely to be female (5/7, 71% vs 25/47, 53%), have a higher BMI (34.4 kg/m^2^ vs 29.5 kg/m^2^), and report poor or fair health at baseline (3/7, 43% vs 5/47, 11%) compared with individuals who completed the study.

The characteristics of the 54 cancer survivors enrolled in this study are presented in Table 2. The mean age at study enrollment was 69.6 years (SD 4.8, range 60-84 years), 44% (24/54) were male, 24% (13/54) were Hispanic, and 57% (31/54) had graduated from college. Most study participants (44/54, 81%) were overweight or obese, 44% (24/54) reported very good or excellent general health, and 50% (27/54) reported 1 or more comorbidities that limited their general activity. There were no significant differences between groups. Among the participants, 39% (21/54) had been diagnosed as having breast cancer, 30% (16/54) had prostate cancer, and 31% (17/54) had a variety of other cancer types. Most patients (40/53, 75%) had been diagnosed as having local-stage disease. The mean age at diagnosis was 65.2 (SD 4.8) years, and the mean number of years between diagnosis and study enrollment was 4.4 (SD 1.6) years.

**Table 2:** Baseline characteristics of the mobile health intervention study participants.

Characteristic					Combined groups (N=54)			Intervention group: tech support (n=18)			Intervention group: tech support+health coaching (n=18)			Waitlist control group (n=18)
**Sociodemographic characteristics**														
	Age (years), mean (SD)			69.6 (4.8)			69.6 (4.5)			69.1 (4.0)			70.2 (5.9)	
	BMI, mean (SD)			30.1 (5.7)			30.2 (6.0)			29.8 (4.8)			30.4 (6.5)	
	**BMI, n (%)**													
		Normal weight	10 (18)			4 (22)			2 (11)			4 (22)		
		Overweight	21 (39)			6 (33)			9 (50)			6 (33)		
		Obese	23 (43)			8 (44)			7 (39)			8 (44)		
	Male, n (%)			24 (44)			10 (56)			6 (33)			8 (44)	
	**Ethnicity, n (%)**													
		Hispanic	13 (24)			5 (28)			4 (22)			4 (22)		
		Non-Hispanic	41 (76)			13 (72)			14 (78)			14 (78)		
	**Race, n (%)**													
		Non-White	4 (7)			1 (6)			2 (11)			1 (6)		
		White	50 (93)			17 (94)			16 (89)			17 (94)		
	College degree, n (%)			31 (57)			11 (61)			11 (61)			9 (50)	
	**Household income, n (%)**													
		<US $50,000	19 (35)			7 (39)			8 (44)			4 (22)		
		≥US $50,000	32 (59)			10 (56)			9 (50)			13 (72)		
		Missing or refused	3 (6)			1 (6)			1 (6)			1 (6)		
**Health and physical functioning**														
	Ever smoker, n (%)^a^			24 (44)			8 (44)			7 (39)			9 (50)	
	**General health status, n (%)**													
		Fair or poor	8 (15)			4 (22)			2 (11)			2 (11)		
		Good	22 (41)			9 (50)			6 (33)			7 (39)		
		Very good or excellent	24 (44)			5 (28)			10 (56)			9 (50)		
	**Number of comorbidities, n (%)**													
		0-1; does not limit activities	27 (50)			10 (56)			8 (44)			9 (50)		
		1-2; limits activities	16 (30)			6 (33)			5 (28)			5 (28)		
		≥3; limits activities	11 (20)			2 (11)			5 (28)			4 (22)		
	**Self-reported physical function, mean (SD)**													
		Raw score (0-100)	73.7 (20.7)			68.1 (22.4)			77.5 (15.4)			75.6 (23.2)		
		T-score^b^	47.5 (7.9)			45.3 (8.6)			48.9 (5.9)			48.2 (8.9)		
	Short Physical Performance Battery (0-12), mean (SD)			10.7 (1.6)			10.4 (2.2)			11.1 (0.9)			10.7 (1.5)	
**Clinical characteristics**														
	**Cancer type, n (%)**													
		Breast	21 (39)			7 (39)			9 (50)			5 (28)		
		Prostate	16 (30)			7 (39)			3 (17)			6 (33)		
		Other^c^	17 (31)			4 (22)			6 (33)			7 (39)		
	**Stage at diagnosis^d^, n (%)**													
		Local	40 (75)			14 (78)			14 (78)			12 (71)		
		Regional	13 (25)			4 (22)			4 (22)			5 (29)		
	**Treatment received^e^, n (%)**													
		Surgery	42 (78)			13 (72)			13 (72)			14 (78)		
		Chemotherapy	10 (18)			3 (17)			3 (17)			4 (22)		
		Radiation	30 (56)			12 (67)			10 (56)			8 (44)		
		Hormone therapy	12 (22)			2 (11)			6 (33)			4 (22)		
	Time since diagnosis (years), mean (SD)			4.4 (1.6)			4.3 (1.4)			4.2 (1.9)			4.6 (1.4)	
**Other characteristics**														
	**Comfort level with using smartphone, n (%)**													
		Very or extremely comfortable	38 (70)			11 (61)			13 (72)			14 (78)		
		Slightly or not comfortable	16 (30)			7 (39)			5 (28)			4 (22)		
	**activPAL data, mean (SD)**													
		Number of valid wear days^f^	6.7 (0.7)			6.6 (1.0)			6.8 (0.4)			6.7 (0.5)		
		Average awake hours	14.5 (1.0)			14.1 (1.1)			14.6 (0.6)			14.6 (0.9)		

^a^Only 1 participant was currently smoking at baseline.

^b^T-scores represent a linear transformation, normed to the US population, with a mean of 50 (SD 10).

^c^Other cancers include bladder, cervical, colon, endometrium, kidney, lymphoma, or melanoma cancers.

^d^Stage at diagnosis is missing for 1 participant.

^e^Percentages do not add up to 100% because participants may have had more than 1 type of treatment.

^f^Up to the first 7 days of 10 hours or more of awake/wear time were included in the analyses; additional days of wear beyond the first 7 days were excluded.

### Adherence

**Figure 3:**
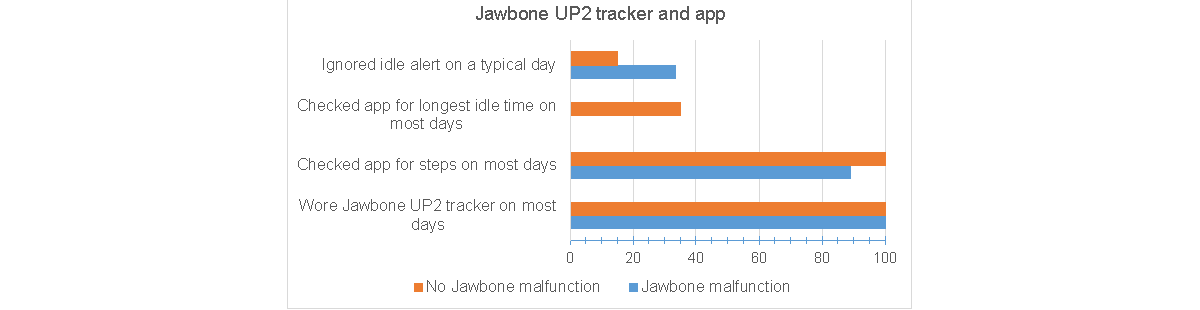
Adherence to wearing the Jawbone UP2 activity tracker and using the smartphone app, stratified by whether the intervention participant experienced malfunctions with the Jawbone UP2 tracker.

Adherence during the intervention was moderately high for wearing the Jawbone activity monitor most days of the week (100% very often) and checking the app daily for the number of steps taken (23/29, 79% very often and 5/29, 17% often; Figure 3). However, few participants checked the app for the longest *idle time* (aka longest sedentary bout; 7/29, 24% often or very often), and on a typical day, most participants ignored the vibration on their tracker and remained seated when reminded to stand up and move (18/29, 62% sometimes and 6/29, 21% often or very often). As indicated in Figure 3, adherence related to the sedentary features of the tracker and app was lower in participants who experienced malfunctions with their initial Jawbone UP2 monitor. Among the participants who completed the trial, 93% (27/29) completed all 5 coaching calls.

### Adverse Events

There were no serious adverse events attributable or possibly attributable to the intervention.

### Acceptability

**Figure 4:**
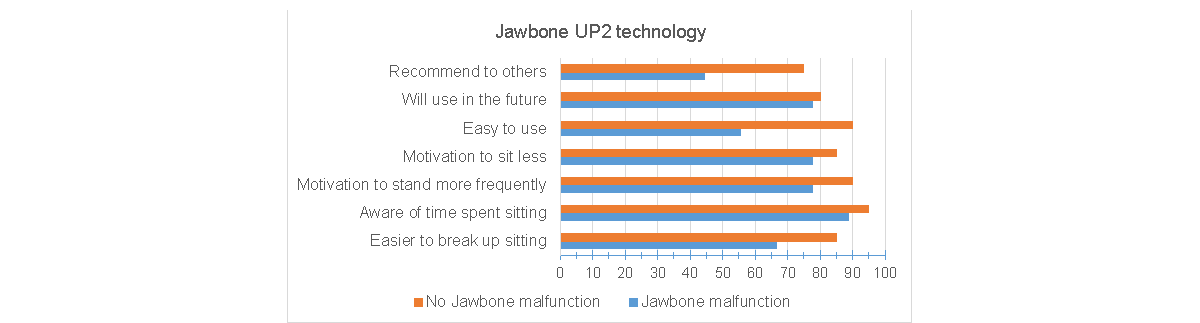
Acceptability and participant evaluation of the mobile health intervention using the Jawbone UP2 activity tracker and smartphone app to sit less, stand more, and move more, throughout the day, and every day. Results are stratified by whether the intervention participant experienced malfunctions with the Jawbone UP2 tracker.

Despite initial Jawbone UP2 malfunctions among one-third of the intervention group, the acceptability of the intervention was moderately high (Figure 4). Overall, 79% (23/29) of the participants agreed or strongly agreed that the Jawbone UP2 technology (monitor plus app) was easy to use and the same percentage indicated that they would use the Jawbone UP2 in the future. Despite the lack of tracking of sedentary data, most participants agreed or strongly agreed that this technology made them more aware of how much time they spent sitting and motivated them to decrease their sedentary time (27/29, 93% and 24/29, 83%, respectively). Participants who started with a malfunctioning Jawbone tracker reported lower acceptability scores than those with properly functioning trackers, with the greatest difference related to ease of use and recommending the tracker and app to others.

### Efficacy Primary Outcomes

Of the 54 cancer survivors enrolled in the study, data for the primary and secondary outcomes for sedentary behavior and physical activity were available for 53 participants (1 monitor malfunction at baseline). On average, participants wore the activPAL monitor for 6.7 days (SD 0.7, range 3-7 days), for an average of 14.5 (SD 1.0) awake/wear hours per day. During a standardized 15 hour awake/wear day, study participants spent 9.6 hours (SD 1.7 h) in sedentary (sitting/lying) activities. Approximately half (5.1, SD 1.7 h) of the number of sedentary minutes were spent in prolonged bouts (30 minutes or longer). The average number of breaks from sitting was 46.6 (SD 14.0) per 15 hour day. Standing accounted for one-quarter of the awake hours (3.8, SD 1.5 h). The remaining time was spent in light- and moderate-intensity stepping (36.8, SD 14.8 minutes and 56.5, SD 25.5 min, respectively; zero minutes in vigorous-intensity stepping). At baseline, only 5 participants met the physical activity guidelines that were recommended at the time the study began (150 minutes per week of moderate-intensity or 75 minutes of vigorous-intensity physical activity, minimum bout duration of 10 min) [17]. On the basis of current guidelines, which no longer require that activity occurs in bouts of at least 10 minutes, 46 participants met the minimum recommendation of at least 150 minutes per week of moderate-intensity activity [88,89].

Between- and within-group comparisons of changes in sedentary behavior are presented in Table 3. The tech support and the tech support plus health coaching groups did not reduce their daily sedentary time compared with the control group (least square means 8.5 min, 95% CI −50.5 to 67.5; *P*=.77 and least square means 10.4 min, 95% CI −43.5 to 64.3; *P*=.70, respectively). There were no significant differences between the intervention and control groups in the daily number of breaks from sitting (least square means −0.1, 95% CI −7.6 to 7.4; *P*=.97 and least square means −2.2, 95% CI −9.0 to 4.7; *P*=.52, respectively). There were no significant or meaningful changes in these sedentary behavior outcomes within any of the 3 groups.

**Table 3:** Between- and within-group comparisons of change in sedentary behavior and physical activity after a 13-week mobile health intervention.^a,b^

Sedentary behavior and physical activity metrics		Baseline, least square mean (95% CI)	Follow-up, least square mean (95% CI)	Within-group change, least square mean difference (95% CI)	*P* value	Between-group change^c^, least square mean difference (95% CI)	*P* value
**Sedentary, minutes per 15 hours awake**							
	Tech support	598.5 (550.1 to 646.9)	604.6 (549.1 to 660.0)	6.0 (−39.5 to 51.6)	.79	8.5 (−50.5 to 67.5)	.77
	Health coaching	567.7 (517.9 to 617.5)	575.6 (525.0 to 626.1)	7.9 (−30.8 to 46.6)	.68	10.4 (−43.5 to 64.3)	.70
	Control	555.4 (507.0 to 603.8)	552.9 (503.8 to 602.0)	−2.5 (−40.0 to 35.0)	.89	N/A^d^	N/A
**Prolonged sedentary bouts (≥30 min), minutes per 15 hours awake**							
	Tech support	319.8 (258.8 to 380.8)	331.9 (263.8 to 400.0)	12.1 (−38.2 to 62.4)	.63	4.7 (−60.3 to 69.7)	.88
	Health coaching	287.0 (224.2 to 349.7)	305.5 (242.0 to 369.0)	18.5 (−23.9 to 61.0)	.38	11.2 (−48.0 to 70.3)	.71
	Control	289.7 (228.7 to 350.7)	297.1 (235.4 to 358.8)	7.4 (−33.8 to 48.6)	.72	N/A	N/A
**Breaks from sitting, number per 15 hour awake**							
	Tech support	50.6 (44.2 to 57.1)	50.5 (43.2 to 57.9)	−0.1 (−5.9 to 5.8)	.97	−0.1 (−7.6 to 7.4)	.97
	Health coaching	48.8 (42.2 to 55.4)	46.6 (39.9 to 53.4)	−2.2 (−7.1 to 2.7)	.38	−2.2 (−9.0 to 4.7)	.52
	Control	46.2 (39.7 to 52.6)	46.2 (39.6 to 52.7)	0.0 (−4.7 to 4.8)	1.00	N/A	N/A
**Break ratio, number of breaks per sedentary hour**							
	Tech support	5.4 (4.5 to 6.2)	5.4 (4.5 to 6.4)	0.06 (−0.66 to 0.77)	.87	−0.08 (−1.00 to 0.85)	.87
	Health coaching	5.3 (4.4 to 6.2)	4.9 (4.0 to 5.8)	−0.39 (−0.99 to 0.21)	.20	−0.52 (−1.36 to 0.32)	.22
	Control	5.1 (4.2 to 5.9)	5.2 (4.3 to 6.0)	0.13 (−0.45 to 0.72)	.65	N/A	N/A
**Standing, minutes per 15 hours awake**							
	Tech support	213.9 (174.2 to 253.7)	202.8 (157.6 to 248.0)	−11.2 (−47.4 to 25.0)	.54	−8.7 (−55.6 to 38.2)	.71
	Health coaching	243.0 (202.1 to 283.9)	220.4 (178.9 to 261.8)	−22.6 (−53.3 to 8.1)	.14	−20.1 (−62.9 to 22.6)	.35
	Control	241.9 (202.2 to 281.6)	239.4 (199.2 to 279.7)	−2.5 (−32.3 to 27.3)	.87	N/A	N/A
**Steps per 15 hour awake**							
	Tech support	6686 (5166 to 8206)	7339 (5594 to 9085)	654 (−794 to 2101)	.37	420 (−1456 to 2297)	.65
	Health coaching	6663 (5099 to 8227)	8338 (6749 to 9926)	1675 (444 to 2906)	.009^e^	1441 (−273 to 3156)	.10
	Control	7898 (6378 to 9418)	8132 (6590 to 9674)	233 (−961 to 1428)	.70	N/A	N/A
**Light-intensity physical activity, minutes per 15 hours awake**							
	Tech support	34.4 (27.2 to 41.5)	33.1 (25.4 to 40.9)	−1.2 (−6.0 to 3.6)	.61	−4.2 (−10.4 to 2.0)	.18
	Health coaching	37.3 (29.9 to 44.7)	36.9 (29.5 to 44.4)	−0.3 (−4.4 to 3.7)	.86	−3.3 (−8.9 to 2.3)	.24
	Control	38.8 (31.6 to 45.9)	41.7 (34.5 to 49.0)	3.0 (−0.9 to 6.9)	.13	N/A	N/A
**Moderate-intensity physical activity (MPA), minutes per 15 hours awake**							
	Tech support	53.2 (40.2 to 66.1)	59.5 (44.5 to 74.6)	6.4 (−6.6 to 19.3)	.33	4.6 (−12.2 to 21.4)	.58
	Health coaching	52.1 (38.8 to 65.4)	67.2 (53.7 to 80.8)	15.2 (4.1 to 26.2)	.008^e^	13.4 (−2.0 to 28.8)	.09
	Control	64.0 (51.0 to 76.9)	65.7 (52.6 to 78.9)	1.8 (−9.0 to 12.5)	.74	N/A	N/A
**MPA (guideline bouts), minutes per 15 hours awake**							
	Tech support	5.8 (−3.2 to 14.8)	13.0 (2.2 to 23.8)	7.3 (−3.1 to 17.6)	.17	7.1 (−6.4 to 20.6)	.30
	Health coaching	3.0 (−6.3 to 12.2)	19.7 (10.2 to 29.1)	16.7 (7.8 to 25.7)	<.001^e^	16.6 (4.1 to 29.0)	.01^e^
	Control	12.1 (3.1 to 21.1)	12.3 (3.1 to 21.4)	0.2 (−8.5 to 8.8)	.97	N/A	N/A

^a^Intent-to-treat analyses.

^b^All variables were standardized to a 15-hour awake per wear day before calculating the pre- to postintervention changes.

^c^Comparisons are between each intervention group and the control group.

^d^N/A: not applicable.

^e^Statistically significant (*P*<.05) results.

### Secondary Outcomes

Between- and within-group comparisons of changes in daily steps and time spent stepping are presented in Table 3. Although time spent standing is considered an LPA, it was evaluated separately from the time spent stepping at a light intensity. There were no significant between-group changes in the time spent standing for either intervention group compared with controls (tech support vs control: least square means −8.7 min, 95% CI −55.6 to 38.2; *P*=.71 and health coaching vs control: least square means −20.1 min, 95% CI −62.9 to 22.6; *P*=.35). There were no significant changes in daily steps between the intervention groups and the control group (tech support vs control: least square means 420 steps, 95% CI −1456 to 2297; *P*=.65 and health coaching vs control: least square means 1441 steps, 95% CI −273 to 3156; *P*=.10). There was a borderline significant difference between moderate-intensity stepping in the health coaching group compared with the control group (least square means 13.4 min, 95% CI −2.0 to 28.8; *P*=.09), but there was no difference between the tech support and control groups (least square means 4.6 min, 95% CI −12.2 to 21.4; *P*=.58). The between-group differences for moderate-intensity stepping accumulated in guideline bouts of 10 minutes or longer were least square means of 16.6 minutes (95% CI 4.1-29.0; *P*=.01) and 7.1 minutes (95% CI −6.4 to +20.6; *P*=.30), respectively, for health coaching group vs controls and tech support group vs controls.

The only significant within-group change occurred in the health coaching group. There was a significant increase of 1675 daily steps (95% CI 444-2906; *P*=.009). Although there was no appreciable change in light-intensity stepping, there was a significant increase in moderate-intensity stepping overall and guideline bouts among the health coaching group (least square means 15.2 extra minutes per day, 95% CI 4.1-26.2; *P*=.008 and least square means 16.7 extra minutes per day, 95% CI 7.8-25.7; *P*<.001). There was neither a significant decrease in sedentary time (least square means 7.9 min, 95% CI −30.8 to 46.6; *P*=.68) nor increase in standing (least square means −22.6 min/day, 95% CI −53.3 to 8.1; *P*=.14). There were no significant within-group changes for either the tech support group or the control group.

### QoL Analysis

There were no significant between-group changes in subjectively measured health-related QoL (Multimedia Appendix 4). However, between-group differences of 4 or more points, representing the minimally clinically significant difference for the SF-36 QoL survey [90], occurred in several subscales. For health coaching compared with controls, these scales included general health, role physical, social functioning, and vitality. For tech support compared with controls, these scales included physical function and social functioning (favoring tech support) and mental health and role emotional (favoring controls). No significant or meaningful between- or within-group differences were observed for the FACIT-Fatigue or the PROMIS pain scales.

### Physical Performance

The average baseline scores on the SPPB were relatively high at baseline for each of the 3 groups (tech support: 10.4, health coaching: 11.2, and control: 10.7). There were no significant between-group changes (*P*>.4); the difference between the health coaching and control groups was at the lower limit of the minimally meaningful change for this scale (0.3-0.8 points) [91].

### Additional Analyses

The results of the complete case analyses, including participants with both baseline and follow-up data, did not differ substantially from the intent-to-treat analyses regarding sedentary behavior and physical activity (data not shown). The results of a sensitivity analysis excluding people with fewer than 4 days of valid activPAL data were not appreciably different from the intention-to-treat analyses (data not shown). No significant between-group differences were found in a sensitivity analysis, excluding participants who experienced issues/failures with the Jawbone tracker. The results for tech support versus controls were as follows (least square mean, 95% CI): sedentary time (−28 min, −99 to 43), standing (17 min, −42 to 76), total daily steps (1290 steps, −403 to 2982), and moderate-intensity stepping (13 min, −2 to 28). The results for health coaching versus controls were as follows: sedentary time (10 min, −56 to 76), standing (−18 min, −72 to 36), total daily steps (1102 steps, −460 to 2663), and moderate-intensity stepping (11 min, −3 to 25).

## Discussion

### Principal Findings

This study explored the feasibility, acceptability, and preliminary efficacy of a home-based mHealth intervention to disrupt and replace sedentary time with LPA (standing and stepping) among older cancer survivors. Despite technical issues with one-third of the Jawbone UP2 activity trackers, an mHealth intervention in older cancer survivors was feasible (high retention and adherence) and acceptable. However, although participants reported that the mHealth intervention increased their awareness of sedentary behavior, this did not translate into a reduction in total sedentary time, prolonged sedentary time, or an increase in breaks from sitting in either intervention group.

The lack of a reduction in total sedentary time was an unexpected finding, given ample room for improvement (nearly 10 hours of sedentary time per day at baseline). In contrast, this group of older, primarily retired, cancer survivors was already taking frequent breaks from sitting, averaging 3 breaks per hour. However, despite the average number of hourly breaks, the amount of time spent in prolonged sedentary bouts (≥30 min) was not reduced, suggesting that there is room for improvement in this metric. Only a few studies have reported a significant increase in the number of breaks from sitting [66]. A large proportion of our study participants reported ignoring the *idle alert* on a typical day. Whether this represented a valid opportunity to stand up and move (eg, alerted while watching television) or an inopportune time (eg, eating, driving, or in a social setting) is unknown. Other studies using the Jawbone tracker reported overall acceptability, including the usefulness or interest in continued use of the *idle alert* [92,93]; however, other studies noted that some participants found the *idle alert* very irritating and inaccurate [94].

In our study, both the postintervention evaluation and comments received from many participants during coaching calls support their focus on the step goal. Similar to other activity tracker apps, the predominant tracking features of the Jawbone apps are related to daily steps rather than sedentary behavior, which may have reinforced the step goal. More support for replacing rather than merely disrupting sedentary time with a suggested minimal bout duration may have been more helpful for individuals already taking frequent breaks from sitting. In addition, research suggests that given the automaticity of sedentary behavior, different and more effortful strategies are required to break existing habits compared with forming new habits [95-97].

Additional unexpected findings were the 6-fold higher time spent standing compared with light-intensity stepping (both before and after intervention) and the suggested decrease in standing, especially in the health coaching group (22 fewer minutes per day). Interventions that report LPA separately indicate that cancer survivors spend 2 to 5 hours per day in these activities [59,60,98,99]. In comparison, our study measured, on average, only 30 to 40 minutes per day. This likely involves measurement differences. Importantly, many interventions have not been able to determine the amount of time spent standing, and standing still is often combined with sedentary time. The activPAL monitor, which is worn on the upper thigh and includes both an inclinometer and accelerometer, provides a more accurate measure of sedentary time (sitting or lying) and standing compared with the ActiGraph accelerometer [70,72], which is the gold standard in MVPA research.

Another research challenge is measuring daily steps in a free-living population (vs in a controlled lab setting), especially if all steps are of interest rather than just higher intensity steps (ie, MVPA). In a free-living population measured during awake hours, stepping ranges from slow, intermittent stepping to fast, continuous stepping. The accuracy of step accumulation by research-grade monitors varies according to walking speed (less accurate at slower speeds) and intermittent (less accurate) versus continuous (more accurate) stepping [100,101]. Therefore, slow or intermittent stepping may be classified as standing rather than light-intensity stepping [100,101]. In our study, overall, there was no reduction in sedentary time, which was measured with high accuracy. Instead, the increased step accumulation among each group, especially the health coaching group, likely represents a shift from standing and slow or intermittent stepping to moderate-intensity and continuous stepping.

There was much flexibility allowed to achieve the goals of the study, that is, no minimum bout duration (standing or stepping) or intensity level (stepping) was provided to participants. The results suggest that most of the intervention group participants focused on the step goal rather than standing more frequently. Furthermore, participants self-selected to accumulate steps in longer bouts and at a moderate versus light intensity. However, only the intervention group with additional health coaching (vs only tech support) achieved significant and meaningful increases in the total daily steps and number of moderate-intensity steps. Although the average number of additional daily steps was below the 3000 goal, it is similar to that reported from meta-analyses using consumer wearable activity trackers, which report 400 to 475 additional daily steps [52,102].

### Comparison With Previous Work

On the basis of recent reviews, interventions with a sedentary behavior focus were more effective (greater reduction in sedentary time) than interventions with a focus on increasing MVPA or both increasing MVPA and reducing sedentary time [103,104]. Reviews of interventions with device-based measurement of sedentary behavior (eg, activPAL and ActiGraph) report, on average, a decrease of 35 minutes per day of sedentary time; however, there was significant heterogeneity detected [51,52,102]. Although device-based measures of sedentary behavior are more accurate than self-report measures, there are also differences in accuracy between device-based measures. For example, hip-worn accelerometers estimate sedentary behavior based on lack of movement (eg, <100 counts per minutes on an ActiGraph), whereas thigh-worn monitors base their estimation on posture (eg, activPAL) [105]. As a result, a hip-worn accelerometer cannot distinguish between standing and sedentary time and can overestimate the change in sedentary time if both sitting and standing are reduced.

To date, few interventions have been designed specifically to decrease sedentary behavior in cancer survivors [106]. In contrast to our findings, several studies have reported a reduction in sedentary time among breast, prostate, and colorectal cancer survivors [58-60]. However, our study compares favorably with the increase in daily steps, especially moderate-intensity stepping. Lynch et al [58] designed an RCT to both reduce sedentary behavior and increase MVPA using the Garmin Vivofit activity tracker among 80 breast cancer survivors (mean age 62 years, SD 6.4). They reported a 37 minutes per day decrease in sitting (95% CI −72.0 to −2.0), which was primarily replaced with standing (27 minutes; 95% CI −2 to 56), and an increase of 933 steps per day (95% CI −215 to 2082). Gomersall et al [59] designed a text-message enhanced clinical exercise intervention (RCT) to reduce sitting time and increase activity among 36 participants, representing several cancer types, primarily colorectal and prostate cancer. The significant decrease in total daily sitting (mean difference −48 minutes/16 h awake day; 95% CI −90 to −6) was primarily replaced with standing (mean difference 42 minutes; 95% CI −4 to 88) and light-intensity stepping (mean difference 7.0 minutes; 95% CI 0.4-14). The RiseTx web-based program designed by Trinh et al [60] included 46 prostate cancer survivors (mean age 73.2 years, SD 7.3) who were given a Jawbone UP 24 activity monitor (model preceding the UP2). The goal was to increase daily steps by 3000 and to reduce sedentary time over a 12-week period in a single-arm trial. There was a significant decrease in sitting time (−455.4 minutes per week; 95% CI −766.6 to −144.2), a nonsignificant decrease in LPA (−91.0 minutes per week; 95% CI −236.4 to 54.4), and a significant increase in MVPA (44.1 minutes per week; 95% CI 11.1-77.0; all measured with the hip-worn ActiGraph). There was also an increase in daily steps (1535; *P*<.001), which was measured using the Jawbone wearable activity tracker rather than a research-grade accelerometer.

### Limitations and Strengths

The limitations of our feasibility study include the potential for selection bias because smartphone ownership was an eligibility criterion. Individuals not familiar with a smartphone (if provided with a loaner phone) may have had more difficulty with adherence or uptake of an mHealth intervention. In addition, individuals who were enrolled were likely more motivated to change their inactivity. The results of this study may not be generalizable to cancer survivors who are less healthy, less physically active, or less comfortable with smartphones than those enrolled in the study. Recruitment was more challenging than anticipated, resulting in a low response rate. Another limitation is the lack of fidelity measures to ensure that the intervention components were delivered as intended. The use of a consumer activity monitor, in this case the Jawbone UP2, is both a limitation and a strength. We experienced substantial technical issues/failures with the device, affecting one-third of the intervention group, as the manufacturing company quit the production, stopped providing support, and eventually closed. While adversely affecting intervention delivery (starting over with tech support/health coaching calls) and possibly retention (3 of 7 dropouts had issues with their Jawbone UP2 monitor; all tech support group), the intervention acceptability scores were moderately high. Most importantly, as reported during follow-up interviews, many intervention participants switched to a different consumer activity monitor to track their steps (Fitbit or Garmin), suggesting a transfer of knowledge and skills gained during the intervention. The strengths of this study include the RCT design and a diverse study sample in terms of sociodemographics, cancer type, and health characteristics. Another strength is the measurement of sedentary behavior with the activPAL research-grade monitor, which is the gold standard for distinguishing between sitting, standing, and stepping [69-72].

Several lessons were learned from this pilot study. First, despite the tremendous growth in the consumer wearable activity tracker market, the disadvantages of using these devices for research studies include technical issues/failures, changes in availability, changes in the user interface or algorithms behind the app, and the potential lack of support from the manufacturer. However, this mHealth approach has been popular among researchers because of its low cost, the ability to reach a large number of participants, and the potential for maintenance of behavior change. The advantages for participants include receiving feedback in real time to prompt change and reducing the burden of tracking weekly/monthly steps (eg, participant recording steps in diary vs automated recording and tracking with app).

Second, sedentary behavior is a strongly ingrained habit that is mostly initiated subconsciously [94]. Research suggests that, given the automaticity of sedentary behavior, different and more effortful strategies are required to break existing habits compared with forming new habits [95-97]. This may require different or multiple behavioral theories to inform the intervention. Although many consumer activity trackers have several behavioral change techniques built into the tracker and/or the app, including Jawbone [54,55,107], accumulating evidence suggests that additional behavior change techniques are needed to achieve meaning change [92,102]. Until activity tracker apps advance to provide features for tracking daily sedentary behavior, researchers will need to provide participants with other strategies. Finally, the daily step goal (+3000 steps above baseline) may have been too high, although participants were able to self-select the minimum bout duration and intensity level for stepping. Nevertheless, the step goal may have competed with messaging to reduce sedentary time.

### Conclusions

This low-touch, home- and technology-based intervention designed to disrupt and replace sedentary time with LPA (standing and stepping) was feasible and acceptable for a diverse group of older cancer survivors. Future studies are warranted to evaluate strategies for replacing sedentary time with standing and/or physical activity.

## Appendix

Multimedia Appendix 1 An example of the activPAL monitor summary data from baseline discussed with intervention group participants in an mHealth study.

Multimedia Appendix 2 activPAL3 data collection and processing details.

Multimedia Appendix 3 CONSORT (Consolidated Standards of Reporting Trials)-EHEALTH checklist (v 1.6).

Multimedia Appendix 4 Effects of mHealth intervention on health-related Quality of Life.

## Abbreviations

CONSORT: Consolidated Standards of Reporting Trials
LPA: light-intensity physical activity
MET: metabolic equivalent
mHealth: mobile health
MVPA: moderate-to-vigorous physical activity
NIH: National Institutes of Health
QoL: quality of life
RCT: randomized controlled trial
SF-36: Short Form 36-item survey
SPPB: Short Physical Performance Battery
