# Supplementary figures and images for: A Home-Based Mobile Health Intervention to Replace Sedentary Time With Light Physical Activity in Older Cancer Survivors: Randomized Controlled Pilot Trial
Source: JMIR Cancer. 2021 Apr 13;7(2):e18819. doi: 10.2196/18819 (PMC8087341; doi:10.2196/18819)

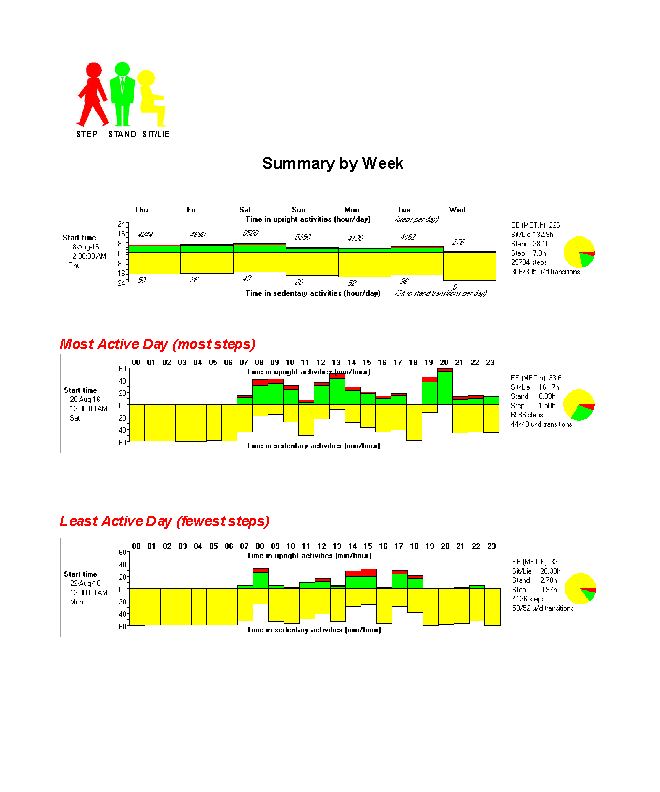

Supplement: Multimedia Appendix 1 [file cancer_v7i2e18819_app1.png]
