# Supplementary material for: A Home-Based Mobile Health Intervention to Replace Sedentary Time With Light Physical Activity in Older Cancer Survivors: Randomized Controlled Pilot Trial
Source: JMIR Cancer. 2021 Apr 13;7(2):e18819. doi: 10.2196/18819 (PMC8087341; doi:10.2196/18819)
Supplement: Multimedia Appendix 2 [file cancer_v7i2e18819_app2.docx]

**Supplemental Table 1. ActivPAL3 data collection and processing details**

| **Item** | **Details** |
| --- | --- |
| Monitor Version | activPAL3^TM^ micro |
| Rationale for selecting activPAL monitor | The primary outcomes, and several secondary outcomes of the feasibility study included sedentary behavior metrics. This monitor is the gold-standard for sedentary behavior research.[1-4] |
| Reliability | Interdevice reliability ranges from 0.79 to 0.99.[3] |
| Validity | “A mean percentage difference of 0.19% (limits of agreement from −0.68% to 1.06%) and 1.4% (limits of agreement from −6.2% to 9.1%) between the activPAL monitor and observation for total time spent sitting and standing has been reported”[3, 5] |
| Method and location of monitor attachment | The 24-hour protocol was used. The activPAL was placed in a nitrile finger cot and then wrapped with Tegaderm dressing to waterproof the device. After watching a visual demonstration, participants attached the device to their right thigh, one-third of the way down between the top of the thigh and top of the knee.[5] Prior to attachment, the participant cleaned the area with an alcohol wipe. The waterproofed activPAL device was attached with Tegaderm dressing. Participants were given extra alcohol wipes and Tegaderm dressing for use during the 7 days of data collection. |
| Wear period and number of days | 24 hours per day for 7 days |
| activPAL software version | 7.2 |
| Setting used: |  |
| Sampling frequency | 20 Hz (default) |
| Minimum sitting period | 10 seconds (default) |
| Minimum upright period | 10 seconds (default) |
| Diary data collected and details collected | Time activPAL attached, Time to bed, time out of bed, time monitor removed, time monitor reattached, and final time activPAL removed after testing |
| Type of file used for data processing | activPAL events file (X, Y, Z version) |
| Method(s) for estimating wearing time/removing time/time in bed/sleep | The sleep/wear logs were manually/visually compared with activPAL generated charts/graphs using PAL Technologies software. The charts include minute by minute information on sitting/lying, standing, and stepping for each hour of each day. To determine/verify time into bed, the following algorithm was used:   - If self-reported time is within an upright event, then time into bed = start of next sedentary event - Else if self-reported time is within a sedentary event AND >15 minutes before next upright event, then time into bed = start time of sedentary event - Else if self-reported time is within a sedentary event AND ≤15 minutes of next upright event, then time into bed = start of next sedentary event   To determine/verify time out of bed, the following algorithm was used:   - If self-reported time is within a sedentary event, then out of bed time = end of sedentary event - Else if self-reported time is within upright event, then out of bed time = end of previous sedentary event   The wear/non wear time and sleep/wake time were entered into an Excel file for each day, per participant, per visit to be used in the data processing package — activPAL Processing R Package.[6] See Appendix 1: Detailed instructions to apply activpalProcess R package.[7] |
| Quality control checks | The participant completed sleep/wear logs were compared manually/visually with the activPAL generated charts/graphs. |
| Action taken for invalid data | Invalid days were excluded from analyses |
| Compliance criteria to define a valid day of observation | Minimum of 10 awake/wear hours per day; ability to determine time out of bed and time in bed |
| Number and type of days required for inclusion in analyses | One or more valid days of activPAL data per intent-to-treat principle |
| Data processing package used and methods used to generate key summary variables | The activPAL Processing R Package (v 1.0.2) was used to process the activPAL events files and calculate the sedentary behavior and physical activity metrics.[6, 7] Instructions to apply activpalProcess R package were followed per Lyden et al.[7] R functions within this package were used to calculate the following:   - total time spent sitting/lying per day - absolute number of breaks from sitting/lying - minutes in sitting/lying bouts that last at least 30 minutes - total time spent standing per day - total number of steps per day - total time in light intensity (1.5-2.99 METs) activity per day - total time in moderate intensity (3.0-5.99 METs) activity per day - total time in vigorous intensity (≥6.0 METs) activity per day - minutes in bouts of MVPA that qualify towards meeting the (older) physical activity guidelines   The Excel file with these metrics was imported into SAS 9.3 for analyses. |

1. Chastin SF, Granat MH. Methods for objective measure, quantification and analysis of sedentary behaviour and inactivity. Gait Posture. 2010 Jan;31(1):82-6. PMID: 19854651. doi: 10.1016/j.gaitpost.2009.09.002.

2. Kozey-Keadle S, Libertine A, Lyden K, Staudenmayer J, Freedson PS. Validation of wearable monitors for assessing sedentary behavior. Med Sci Sports Exerc. 2011 Aug;43(8):1561-7. PMID: 21233777. doi: 10.1249/MSS.0b013e31820ce174.

3. Grant PM, Ryan CG, Tigbe WW, Granat MH. The validation of a novel activity monitor in the measurement of posture and motion during everyday activities. Br J Sports Med. 2006 Dec;40(12):992-7. PMID: 16980531. doi: 10.1136/bjsm.2006.030262.

4. Sellers C, Dall P, Grant M, Stansfield B. Validity and reliability of the activPAL3 for measuring posture and stepping in adults and young people. Gait Posture. 2016 Jan;43:42-7. PMID: 26669950. doi: 10.1016/j.gaitpost.2015.10.020.

5. Edwardson CL, Winkler EAH, Bodicoat DH, Yates T, Davies MJ, Dunstan DW, et al. Considerations when using the activPAL monitor in field-based research with adult populations. J Sport Health Sci. 2017 Jun;6(2):162-78. PMID: 30356601. doi: 10.1016/j.jshs.2016.02.002.

6. Lyden K. Package 'activpalProcessing'. Available from: <https://cran.r-project.org/web/packages/activpalProcessing/index.html>.

7. Lyden K, Keadle SK, Staudenmayer J, Freedson PS. The activPALTM Accurately Classifies Activity Intensity Categories in Healthy Adults. Med Sci Sports Exerc. 2017 May;49(5):1022-8. PMID: 28410327. doi: 10.1249/MSS.0000000000001177.
