# Supplementary material for: A Home-Based Mobile Health Intervention to Replace Sedentary Time With Light Physical Activity in Older Cancer Survivors: Randomized Controlled Pilot Trial
Source: JMIR Cancer. 2021 Apr 13;7(2):e18819. doi: 10.2196/18819 (PMC8087341; doi:10.2196/18819)
Supplement: Multimedia Appendix 4 [file cancer_v7i2e18819_app4.docx]

**Supplemental Table 2 Effects of mHealth intervention on health-related Quality of Life**

|  | **Baseline** | **Follow-up** | **Within-group change** | **P** | **Between group change** | **p** |
| --- | --- | --- | --- | --- | --- | --- |
| **SF-36 Quality of Life^a^** |  |  |  |  |  |  |
| **Bodily Pain** |  |  |  |  |  |  |
| Tech Support | 60.2 (49.6, 70.8) | 61.6 (49.3, 73.9) | 1.4 (-10.4, 13.2) | 0.81 | -3.7 (-19.2, 11.8) | 0.63 |
| Health Coaching | 62.1 (51.2, 73.0) | 68.4 (56.7, 80.1) | 6.3 (- 4.8, 17.4) | 0.26 | 1.2 (-13.8, 16.2) | 0.87 |
| Control | 59.1 (48.5, 69.6) | 64.2 (53.6, 74.8) | 5.1 (- 4.9, 15.1) | 0.31 |  |  |
|  |  |  |  |  |  |  |
| **General Health** |  |  |  |  |  |  |
| Tech Support | 62.8 (54.3, 71.3) | 64.7 (55.6, 73.7) | 1.8 (- 3.8, 7.4) | 0.51 | 3.3 (- 4.0, 10.5) | 0.37 |
| Health Coaching | 70.2 (61.4, 79.0) | 73.9 (65.1, 82.7) | 3.7 (- 1.2, 8.6) | 0.13 | 5.1 (- 1.6, 11.8) | 0.13 |
| Control | 71.5 (63.0, 80.0) | 70.1 (61.5, 78.6) | -1.4 (- 6.1, 3.2) | 0.53 |  |  |
|  |  |  |  |  |  |  |
| **Mental Health** |  |  |  |  |  |  |
| Tech Support | 78.1 (70.4, 85.7) | 77.3 (68.8, 85.8) | -0.7 (- 7.4, 6.0) | 0.83 | -5.2 (-14.0, 3.5) | 0.23 |
| Health Coaching | 75.3 (67.4, 83.2) | 79.4 (71.3, 87.5) | 4.1 (- 2.0, 10.2) | 0.18 | -0.4 (- 8.7, 7.8) | 0.92 |
| Control | 75.6 (67.9, 83.2) | 80.1 (72.4, 87.7) | 4.5 (- 1.1, 10.1) | 0.11 |  |  |
|  |  |  |  |  |  |  |
| **Physical Function** |  |  |  |  |  |  |
| Tech Support | 68.1 (58.1, 78.0) | 72.0 (61.0, 82.9) | 3.9 (-4.2, 12.0) | 0.33 | 4.5 (-6.1, 15.0) | 0.40 |
| Health Coaching | 76.5 (66.2, 86.8) | 76.8 (66.4, 87.2) | 0.3 (-6.8, 7.5) | 0.93 | 0.9 (-8.9, 10.7) | 0.86 |
| Control | 75.6 (65.6, 85.5) | 75.0 (65.0, 85.0) | -0.6 (-7.3, 6.2) | 0.87 |  |  |
|  |  |  |  |  |  |  |
| **Role Emotional** |  |  |  |  |  |  |
| Tech Support | 79.6 (68.6, 90.7) | 83.8 (71.0, 96.5) | 4.1 (-7.8, 16.0) | 0.49 | -4.7 (-20.2, 10.9) | 0.55 |
| Health Coaching | 82.4 (71.0, 93.8) | 87.5 (75.9, 99.2) | 5.2 (-5.4, 15.8) | 0.33 | -3.6 (-18.2, 11.0) | 0.62 |
| Control | 80.6 (69.5, 91.6) | 89.4 (78.3, 100) | 8.8 (-1.3, 18.9) | 0.09 |  |  |
|  |  |  |  |  |  |  |
| **Role Physical** |  |  |  |  |  |  |
| Tech Support | 68.1 (55.7, 80.4) | 69.6 (55.9, 83.3) | 1.5 (-9.3, 12.4) | 0.78 | 1.9 (-12.3, 16.0) | 0.79 |
| Health Coaching | 69.5 (56.8, 82.2) | 73.5 (60.7, 86.4) | 4.1 (-5.5, 13.6) | 0.40 | 4.4 (- 8.8, 17.6) | 0.50 |
| Control | 70.1 (57.8, 82.5) | 69.8 (57.5, 82.1) | -0.3 (-9.4, 8.7) | 0.94 |  |  |
|  |  |  |  |  |  |  |
|  |  |  |  |  |  |  |
| **Social Functioning** |  |  |  |  |  |  |
| Tech Support | 80.6 (69.8, 91.3) | 80.9 (69.2, 92.7) | 0.4 (- 8.1, 8.8) | 0.93 | 5.9 (-5.1, 16.9)_ | 0.28 |
| Health Coaching | 81.6 (70.5, 92.7) | 84.3 (73.0, 95.7) | 2.7 (- 5.0, 10.3) | 0.48 | 8.2 (-2.1, 18.6) | 0.12 |
| Control | 88.2 (77.4, 99.0) | 82.6 (71.9, 93.4) | -5.6 (-12.6, 1.5) | 0.12 |  |  |
|  |  |  |  |  |  |  |
| **Vitality** |  |  |  |  |  |  |
| Tech Support | 61.8 (52.1, 71.5) | 61.7 (51.0, 72.4) | -0.1 (-8.3, 8.2) | 0.99 | -0.1 (-10.8, 10.7) | 0.99 |
| Health Coaching | 56.3 (46.3, 66.2) | 60.8 (50.6, 71.1) | 4.6 (-2.9, 12.1) | 0.22 | 4.6 (- 5.6, 14.8) | 0.37 |
| Control | 65.3 (55.6, 75.0) | 65.3 (55.6, 75.0) | 0 (-6.0, 6.9) | 1.0 |  |  |
|  |  |  |  |  |  |  |
| **FACIT Fatigue^b^** |  |  |  |  |  |  |
| Tech Support | 37.7 (33.8, 41.7) | 38.3 (34.1, 42.5) | 0.6 (-2.1, 3.2) | 0.67 | -0.7 (-4.1, 2.7) | 0.68 |
| Health Coaching | 36.5 (32.5, 40.6) | 38.0 (33.9, 42.0) | 1.4 (-0.9, 3.7) | 0.22 | 0.2 (-3.0, 3.3) | 0.92 |
| Control | 36.3 (32.3, 40.2) | 37.5 (33.6, 41.5) | 1.3 (-0.9, 3.4) | 0.25 |  |  |
|  |  |  |  |  |  |  |
| **PROMIS Pain^c^** |  |  |  |  |  |  |
| Tech Support | 51.6 (47.4, 55.8) | 55.4 (50.4, 60.5) | 3.8 (-1.1, 8.7) | 0.12 | 3.1 (-3.2, 9.4) | 0.32 |
| Health Coaching | 52.5 (48.2, 56.8) | 51.2 (46.8, 55.6) | -1.3 (-5.5, 2.9) | 0.53 | -2.0 (-7.8, 3.8) | 0.49 |
| Control | 51.0 (46.8, 55.1) | 51.6 (47.5, 55.8) | 0.7 (-3.3, 4.7) | 0.73 |  |  |
|  |  |  |  |  |  |  |
| **Short Physical Performance Battery (SPPB)^d^** |  |  |  |  |  |  |
| Tech Support | 10.4 ( 9.7, 11.2) | 10.7 ( 9.9, 11.5) | 0.2 (-0.5, 1.0) | 0.50 | 0.2 (-0.8, 1.1) | 0.69 |
| Health Coaching | 11.2 (10.4, 11.9) | 11.5 (10.8, 12.3) | 0.4 (-0.3, 1.0) | 0.26 | 0.3 (-0.6, 1.2) | 0.49 |
| Control | 10.7 ( 9.9, 11.4) | 10.7 (10.0, 11.5) | 0.1 (-0.5, 0.7) | 0.85 |  |  |
|  |  |  |  |  |  |  |

^a^ The eight subscales of the SF-36 QOL survey represent raw scores ranging from 0 to 100. Higher scores represent better QOL.

^b^ The FACIT Fatigue scale includes 13 questions on whether and the degree to which fatigue had affected a person’s life during the past 7 days.

^c^ The PROMIS pain interference survey includes 8 questions on whether and the degree to which pain had interfered with various activities during the past 7 days.

^d^ The SPPB score ranges from 0 to 12 with higher numbers indicating better physical performance
